# Mechanical and Physical Characterization of a Biphasic 3D Printed Silk-Infilled Scaffold for Osteochondral Tissue Engineering

**DOI:** 10.1021/acsbiomaterials.4c01865

**Published:** 2024-11-26

**Authors:** T. Braxton, K. Lim, C. Alcala-Orozco, H. Joukhdar, J. Rnjak-Kovacina, N. Iqbal, T. Woodfield, D. Wood, C. Brockett, X.B. Yang

**Affiliations:** † Biomaterials and Tissue Engineering Group, Department of Oral Biology, University of Leeds, WTBB, St. James’s University Hospital, Leeds LS9 7TF, U.K.; ‡ CReaTE Group, Department of Orthopaedic Surgery, University of Otago Christchurch, Christchurch 8140, New Zealand; § Graduate School of Biomedical Engineering, UNSW Sydney, Sydney, New South Wales 2052, Australia; ∥ Chemical and Process Engineering, University of Leeds, Leeds LS2 9JT, U.K.; ⊥ School of Mechanical Engineering, University of Leeds, Leeds LS2 9JT, U.K.

**Keywords:** Osteochondral, Cartilage regeneration, Tissue Engineering, Silk fibroin, 3D printing, Biphasic scaffold

## Abstract

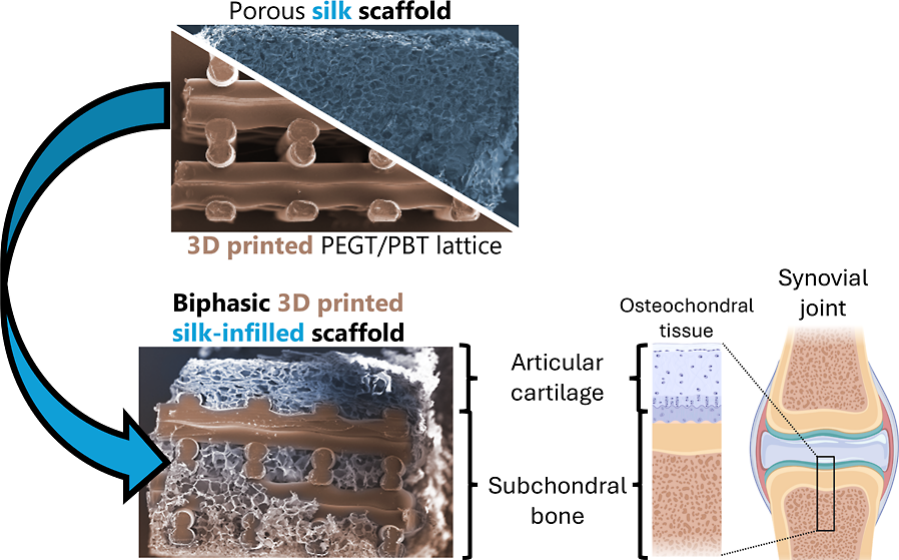

Osteochondral tissue damage is a serious concern, with even minor cartilage damage dramatically increasing an individual’s risk of osteoarthritis. Therefore, there is a need for an early intervention for osteochondral tissue regeneration. 3D printing is an exciting method for developing novel scaffolds, especially for creating biological scaffolds for osteochondral tissue engineering. However, many 3D printing techniques rely on creating a lattice structure, which often demonstrates poor cell bridging between filaments due to its large pore size, reducing regenerative speed and capacity. To tackle this issue, a novel biphasic scaffold was developed by a combination of 3D printed poly(ethylene glycol)-terephthalate-poly(butylene-terephthalate) (PEGT/PBT) lattice infilled with a porous silk scaffold (derived from *Bombyx mori* silk fibroin) to make up a bone phase, which continued to a seamless silk top layer, representing a cartilage phase. Compression testing showed scaffolds had Young’s modulus, ultimate compressive strength, and fatigue resistance that would allow for their theoretical survival during implantation and joint articulation without stress-shielding mechanosensitive cells. Fluorescent microscopy showed biphasic scaffolds could support the attachment and spreading of human mesenchymal stem cells from bone marrow (hMSC-BM). These promising results highlight the potential utilization of this novel scaffold for osteochondral tissue regeneration as well as highlighting the potential of infilling silk materials within 3D printed scaffolds to further increase their versatility.

## 1 Introduction

Osteochondral tissue damage is a serious concern, with even minor cartilage damage increasing an individual’s risk of suffering from joint discomfort or pain and can lead to osteoarthritis (OA).^1,2^ Osteochondral damage is often seen as a result of traumatic injury from sports or work and is particularly prevalent in young active patients.^3^ Articular cartilage demonstrates a poor self-repair capability, with even small-sized lesions failing to heal due to it is unique structure which does not have blood vessels, nerves and lymphatics.^4^ One of the most common treatments for osteoarthritis is joint replacement; however, this intervention is highly invasive and the joint replacement has a limited lifespan.^5^ Therefore, there is a need for an earlier intervention before the onset of OA. Tissue engineering presents a unique opportunity to address this need to regenerate osteochondral tissue. But osteochondral tissue regeneration presents several unique challenges due to its multitissue composition. Osteochondral tissue comprises both articular cartilage and underlying subchondral bone, each tissue type presenting unique challenges for regeneration and requiring a novel approach.

Additive manufacturing through 3D printing has emerged as a highly versatile and cost-effective process, especially in fields like tissue engineering, where it allows for the rapid and personalized creation of complex structures with precise control over bulk geometry.^6^ Recent advancements in 3D printing have revolutionized material science by enabling layer-by-layer construction of intricate geometries, offering unprecedented control over the spatial distribution of materials and internal structures. This process significantly shortens production times while enhancing the ability to fabricate customized scaffolds and optimized designs. Poly(ethylene glycol)-terephthalate-poly(butylene terephthalate) block copolymer (PEGT/PBT) is a series of segmented block copolymers; the properties of this thermoplastic, as with all block copolymers, are a result of its constituent segments blending both their mechanical and physical properties.^7,8^ The relatively soft and hydrophilic nature of the PEGT segments contributes elastomeric properties and hydrophilicity, whereas the hard hydrophobic PBT segments contribute rigidity and improved mechanical strength. Researchers have demonstrated the biocompatibility and biodegradability of PEGT/PBT blends.^9−13^ However, there remains a major issue with the utilization of 3D printed lattice is as biological scaffolds in that the scaffolds suffer from limited cellular migration due to the relatively large distance between the 3D printed filaments leading to the formation of large pores in the scaffold. The substantial size of the pores prevents cells from bridging the gaps efficiently, diminishing their migration ability throughout the scaffold.^14^ The reduction in cellular migration subsequently leads to a reduced rate of tissue regeneration as cells are unable to effectively populate the scaffold and regenerate new tissue. Due to the nature in which the filaments of a 3D-printed lattice scaffold are deposited, the pore size cannot be reduced without reducing scaffold porosity in turn. Therefore, this is not a viable method for reducing scaffold pore size within 3D printed lattice scaffolds. As for adequate tissue regeneration in general, it has been estimated that approximately 70% scaffold porosity is required for adequate cellule infiltration and regeneration^15,16^

In contrast to 3D printing, scaffolds can also be formed into foam or sponge-like structures by using a simple freeze-drying process to form a highly porous scaffold.^17^ The porous nature of these scaffolds gives the physical surface onto which the cells can lay their extracellular matrix (ECM) and gives rise to a large surface area for cell binding and spreading.^18^ Silk fibroin from the silkworm *Bombyx mori* is already used in various biomedical applications. The reason for its extensive use is that silk fulfils many of the requirements for a successful biomaterial; such as possessing biodegradability, biocompatibility, a minimal inflammatory response postimplantation, as well as long-term compatibility, and allows for cell adhesion to its surface.^19−21^ However, porous sponge scaffolds derived from natural polymers are often mechanically weak, particularly when highly porous, thus limiting their ability to withstand mechanical stresses and strains, which can be particularly problematic when used in load-bearing applications. Moreover, natural polymer-based sponge scaffolds can also be rapidly degraded. While a biological scaffold’s ability to be degraded over time is essential to its functionality, rapid degradation can be problematic as it can compromise the mechanical stability of the scaffold and limit its ability to support tissue regeneration over the long-term.^22^

Achieving balance in the scaffold creation process between the scaffold’s load-bearing ability and its success at cellular viability is imperative; This balance can be found by combining the advantageous properties of 3D printed synthetic constructs and natural porous scaffolds. Therefore, in this study, we aimed to create a unique biphasic scaffold consisting of a porous silk cartilage phase, which is seamlessly integrated with a bone phase consisting of a 3D printed lattice infilled with a porous silk sponge for osteochondral tissue regeneration. This study focuses on the characterization of a biphasic 3D printed silk-infilled scaffold physical and mechanical properties as an indication for its potential further use within bone and osteochondral tissue regeneration.

The physical and mechanical properties of a scaffold are crucial indicators of its potential efficacy in osteochondral tissue regeneration. These properties determine the scaffold’s ability to provide appropriate support and structural integrity which is essential for tissue formation and function. Mechanical strength and stiffness must prevent scaffold failure under physiological stresses. Additionally, physical characteristics such as porosity and surface topology influence cell attachment, proliferation, and differentiation, which are vital for the integration of new tissue. Therefore, assessing these properties ensures the scaffold can effectively support tissue regeneration, maintain structural stability, and integrate seamlessly with the host tissue, ultimately leading to successful osteochondral repair.

## 2 Materials and Methods

### 2.1 Scaffold Design and Fabrication

The 3D printed PEGT/PBT scaffold (15 × 15 × 2 mm with a 0.75 mm pore size) scaffolds were printed by 3D BioPlotter (EnvisionTec) at a printing temperature of 180 °C using PEGT/PBT (Polyactive 300PEGT55PBT45, PolyVation, The Netherlands) with a PEG molecular weight (MW) of 300 g mol^–1^ and a PEGT/PBT (55:45 wt %)

The silk fibroin was extracted from *B. mori* cocoons as previously described^23^ Briefly, silk cocoons were degummed in boiling sodium carbonate solution (0.02 M) (Sigma-Aldrich, St. Louis, MO) for 30 min to remove sericin. The pure silk fibroin was then solubilized in a lithium bromide solution (9.3 M) (Sigma-Aldrich) at 60 °C for 4 h at a 20 wt %/v of silk to lithium bromide. Lithium bromide was then removed from the solution via dialysis (3500 MWCO, EMD Millipore) in deionized water for 3 days.

A 5% silk solution was then cast into 3D printed lattices or alone (as the control) into 12 well plates (1.5 mL of silk solution in each well). To improve pore filling by the silk solution over the 3D printed scaffolds, scaffolds were placed under vacuum for 5 min. Following this, samples were frozen overnight at −20 °C followed by lyophilization in a freeze-dryer. Dried constructs were then removed from the plate and wrapped in aluminum foil and autoclaved at 121 °C for 20 min to induce beta-sheet formation in silk and sterilize the constructs. Silk only scaffolds were also created using the same protocol but without the 3D-printed lattice. Before use, scaffolds were cut to 5 mm^2^ constructs and rehydrated overnight by rocking in 1× PBS (Corning 21-040-CV) at room temperature. This was followed by placing them under negative pressure for 5 min while submerging them within 1× PBS (unless stated otherwise).

### 2.2 Characterization of Scaffold Surface Morphology and Pore Size Using Scanning Electron Microscopy

Scaffold morphology was investigated through a scanning electron microscope (SEM) (Hitachi S3400N variable pressure SEM) at various magnifications with an electron exoneration voltage of 10.0–20.0 Kv. Prior to imaging, samples were sputter coated with gold.

Pore size for the silk scaffolds, 3D printed scaffolds and the cartilage and bone phase of biphasic scaffold was determined by taking SEM images (at one hundred times magnification) at three zones of each scaffold (*n* = 4). The mean pore diameter was calculated by manually measuring a minimum of 40 pores per image using ImageJ. software (version 1.41).

### 2.3 Element Analysis of the ScaffoldsUsing Elemental Dispersive X-ray

Energy dispersive X-ray analysis was performed with dual Bruker XFlash detectors attached to a Hitachi S3400N variable pressure SEM. Analysis was undertaken with Quantax analysis software (1.9). The accelerating voltage was set to 10 kV for all EDX measurements. Quantifications were undertaken at 3 distinct and separate locations within 4 separate scaffolds for each group (3D printed control scaffolds, silk control scaffolds, and both the cartilage and the bone phase of the biphasic scaffolds), and the average was taken. Quantax analysis software utilizes a peak-to-background ZAF evaluation (P/B-ZAF) algorithm to quantify the presence of various elements found within the sample. Bremsstrahlung’s background was automatically calculated. A Bayes deconvolution was used for line overlap separation.

### 2.4 Scaffold Porosity Analysis Using the Archimedes Method

Scaffold porosity was calculated as per the Archimedes method.^24^ Silk control scaffolds, biphasic scaffolds and 3D printed scaffolds (*n* = 4) were preweighed, followed by rehydration in ethanol under negative pressure for 5 min. The scaffolds were removed, excess liquid was removed using filter paper, and weight was recorded. The scaffolds were then resubmerged in ethanol, and the submerged weight was measured. Scaffold porosity was then calculated.
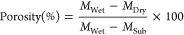
where *W*_Dry_ is the dry weight of scaffolds, *W*_Wet_ is the weight of the scaffold after hydration in ethanol, and *W*_Sub_ is the weight of the scaffolds submerged in ethanol.

### 2.5 Mechanical Characterization of the Scaffolds with Uniaxial Compression Testing

Load-to-failure uniaxial compression testing was utilized in wet unconfined conditions to determine the structural integrity of biphasic 3D printed silk-infilled scaffold scaffolds (*n* = 4) and silk scaffolds (*n* = 6). Prior to mechanical testing, all scaffolds were rehydrated in 1 × PBS for 24 h, followed by negative pressure rehydration to confirm complete rehydration. Uniaxial compression testing was used to measure the ultimate compressive strength and Young’s modulus. The biphasic and 3D printed scaffold were tested until failure with a 500 N load cell at a strain rate of 0.1 mm min^–1^ (Instron 3365). Silk scaffolds were tested until the maximum load of the testing instrument on a 8 N load cell at a strain rate of 0.1 mm min–1 (Bose ElectroForce 3200 Series III Test Instrument). Fatigue testing was utilized to determine the long-term resistance to mechanical loading. Biphasic scaffolds (*n* = 3), silk scaffolds (*n* = 4) and 3D printed scaffolds (*n* = 4) were subjected to a load of 8 N at 100,000 cycles with a 1 Hz sinusoids wave pattern on a Bose ElectroForce 3200 Series III Test Instrument with a 8 N load cell. The maximum induced strain was recorded every 100 cycles, the height of the scaffolds after testing was determined, and the percent reduction in height was calculated.
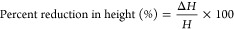
where *H* is the original height of the sample, and Δ*H* is the change in height of the sample.

### 2.6 Swelling Capacity and Degradation Analysis of Scaffolds

The swelling capacity of the scaffolds was assessed in 1× PBS. Silk control scaffolds, 3D printed control scaffolds, and biphasic scaffolds (*n* = 4) were preweighed, followed by rehydration in 1× PBS at 37 °C; every 1 h, scaffolds were removed, and excess liquid was removed using filter paper, and weight was recorded. The scaffolds were then returned to fresh 1× PBS. The percentage increase in mass was calculated by comparing the scaffolds’ dry and hydrated mass. After 18 h, scaffolds were submerged in 1× PBS and left under negative pressure for 5 min before being weighed and compared to the mass of the scaffold before negative pressure rehydration.
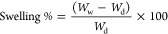
where *W*_w_ and *W*_d_ are the wet and dry weights of the samples, respectively.

Degradation analysis of the scaffold in vitro The initial mass of dry scaffolds was recorded. The scaffolds (*n* = 4 per group) were then placed in preweighted 1.5 mL Eppendorf tubes. One mL of 2 U/mL Protease XIV solution (in 1× PBS) was added to each tube and incubated at 37 °C. The Protease XIV was removed every 2 days. Scaffolds were then washed with deionized water and dried overnight at 60 °C. Dry mass was recorded, the remaining mass percentage was calculated, and fresh Protease XIV solution was added.
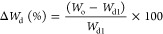
where *W*_o_ and *W*_d1_ refer to the initial sample weight and the sample weight at time (*t*), respectively.

### 2.7 Cells Culture

Human mesenchymal stem cells from bone marrow (hMSC-BM) (PromoCell, C-12974) were cultured in basal expansion medium consisting of the alpha modified minimum essential medium (α-MEM) (Corning 15-012-CV), containing 10% (v/v) fetal bovine serum (FBS) (Sigma-Aldrich, F75240), penicillin/streptomycin (P/S) (100 units/ml, and 100 μg/mL respectively) (Sigma-Aldrich P0781) and 1 ng/mL recombinant human basic fibroblastic growth factor (bFGF) (PeproTech, 100-18B). The medium was changed every 3–4 days. All cells were passaged at approaching approximately 80% confluence and passage 3 cells were used for the experiments accordingly.

### 2.8 Contact Cytotoxicity Assay by Giemsa Staining

Silk control scaffolds, biphasic scaffolds and 3D printed scaffolds were attached to 6 well plates (corning, cat no. 3516) with the aid of steri-strips (Medisave, R1540C), steri-strips alone and 40% dimethyl sulfoxide (DMSO) were used as the positive and negative controls respectively (*n* = 3). For all groups 1× PBS was used to wash the wells twice, aspirated, and 2 mL of hMSC-BM cell suspension containing 50,000 cells was added to each well. The culture plates were incubated at 37 °C for 96 h in 5 (v/v)% CO2 in an incubator. After 96 h, the media was aspirated from the wells and washed twice with 1× PBS. One mL of 10 (v/v)% neutral-buffered formalin (Cellpath, BAF-0010-01A) was added to each well and incubated for 15 min. The formalin was aspirated, and all wells were stained for 5 min using Giemsa solution, then washed using distilled water. The culture plates were air-dried for 24 h and examined microscopically to record any changes in morphology, confluency, attachment, and detachment of the hMSC-BM using a Leica DM16000 B inverted microscope.

### 2.9 Cell Viability

hMSC-BM (500,000 cells per scaffold) were statically seeded onto the 3D-printed scaffolds, silk scaffolds, the cartilage phase of biphasic scaffolds and the bone phase of biphasic scaffolds. All scaffolds were initially rehydrated in 1× PBS for 12 h, followed by negative pressure rehydration for 5 min. Scaffolds were then seeded by submerging in a 1.5 mL basal media containing 500,000 cells per scaffold for 24 h in a standard cell incubator. After 24 h, the cells on the constructs (*n* = 4) were labeled with Cell Tracker Green 5-chloromethyl fluorescein diacetate (CFMDA, ThermoFisher Scientific, C7025) and visualized using a TCS SP8 confocal laser scanning microscope (Leica, Germany).

### 2.10 Statistical Analysis

Statistical analysis was run using SPSS (26) and Microsoft Excel. Data was tested for normality using a Shapiro–Wilk test and QQ plots; all data was found to be normally distributed. A two-tailed T-Test and ANOVA with Bonferroni posthoc tests were performed. *P* ≤ 0.05 was considered as statistically significant.

## 3 Results

**Figure 1:**
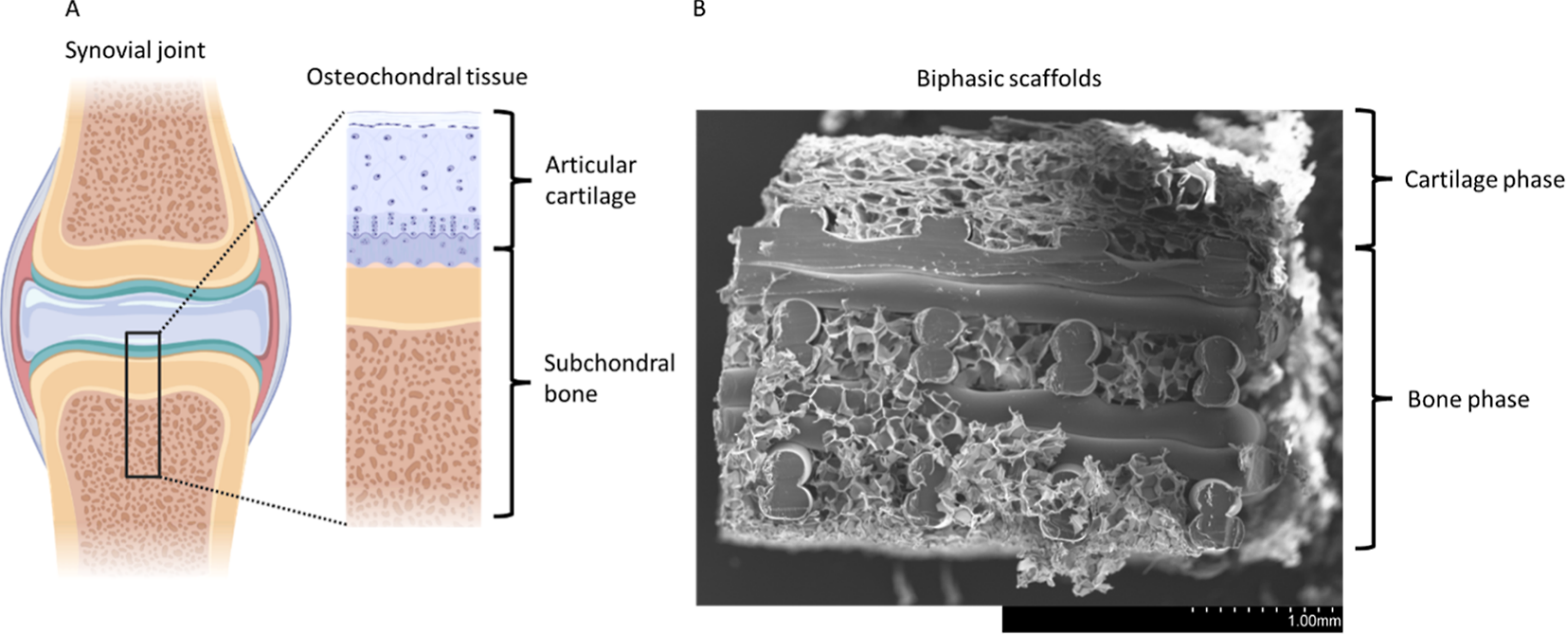
Comparison of a schematic representation of natural osteochondral tissue (A) compared to the morphology of biphasic scaffolds (B), highlighting the location and morphology of cartilage and bone phases, and the composition of the scaffold. This highlights the comparative regions between the designed biphasic scaffold and natural tissue. Figure was created with the assistance of BioRender.com.

Biphasic 3D printed silk-infilled scaffolds (Biphasic scaffolds) were successfully fabricated. Biphasic scaffolds consisted of a flexible and resilient porous silk scaffold (cartilage phase) seamlessly integrated into a mechanically strong silk-infilled 3D printed PEGT/PBT scaffold (bone phase), mimicking the natural stratified structure seen in osteochondral tissue (Figure 1).

### 3.1 Structural Characterization of Different Scaffolds

**Figure 2:**
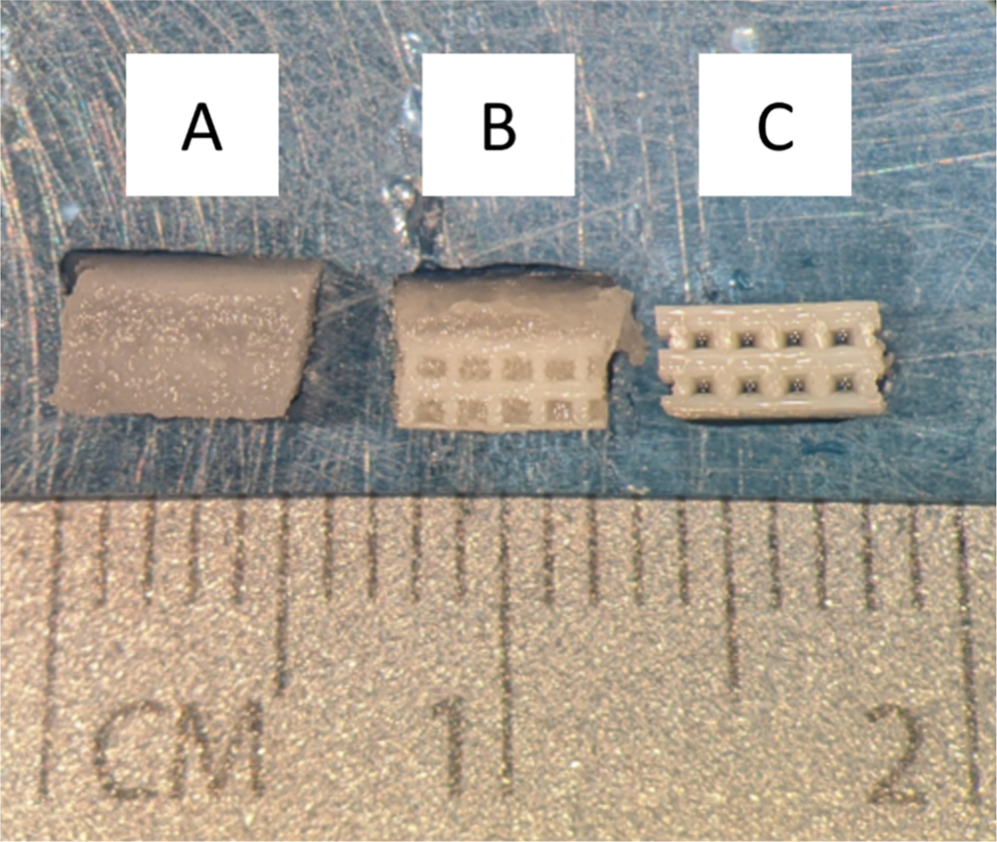
Stereomicroscope image representing the structural characteristics of the different scaffolds: silk scaffolds (A), biphasic scaffolds (B), and 3D printed scaffolds (C).

**Figure 3:**
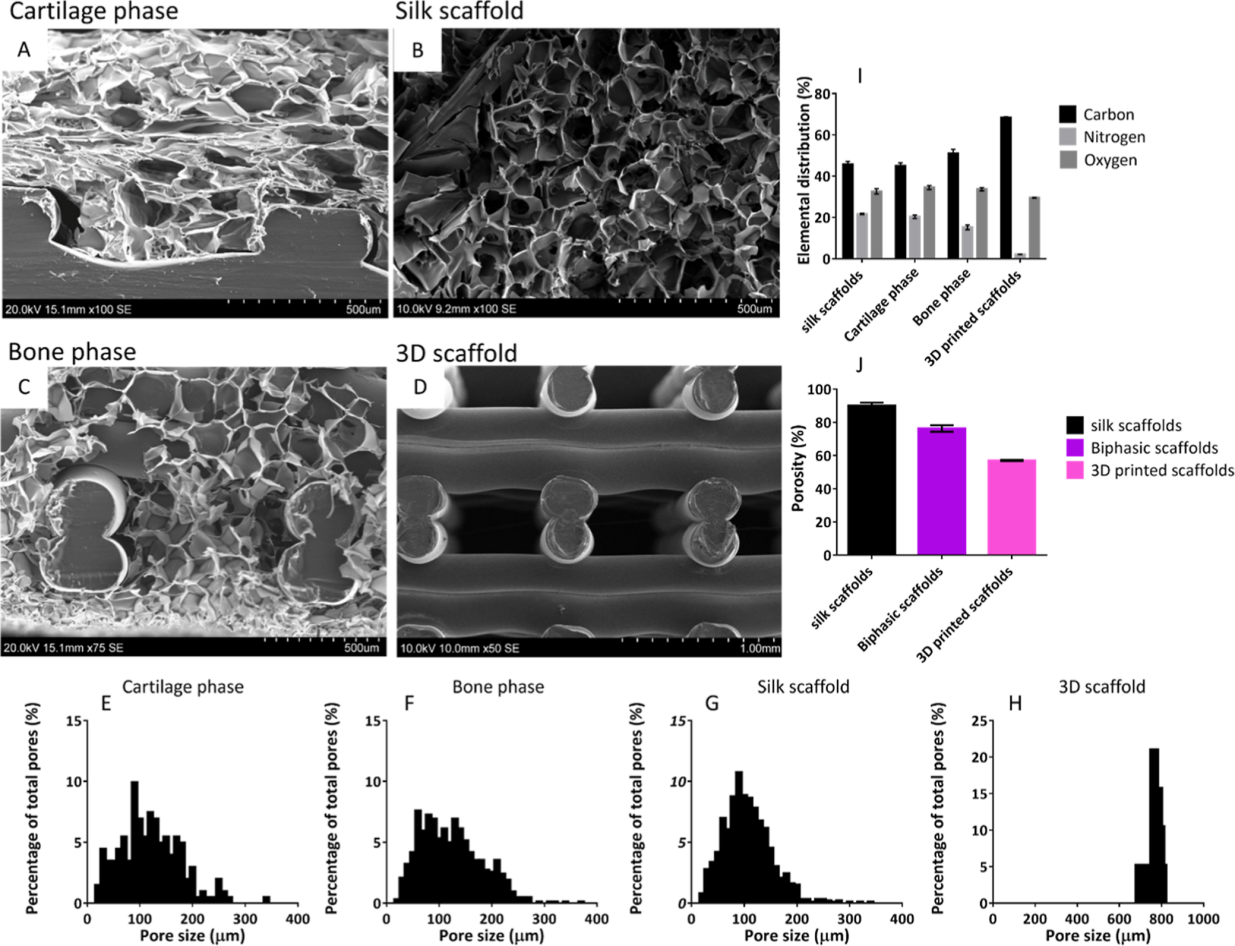
SEM images (A–D) and quantitative measurement (E–J) of the scaffold morphological characteristics. (A) SEM micrograph of the structural morphology of cartilage phase within biphasic scaffolds. (B) SEM micrograph of the structural morphology of bone phase within biphasic scaffolds and the interactions between silk scaffold and 3D printed lattice. (C) SEM micrograph of the structural morphology of silk scaffolds. (D) SEM micrograph of the structural morphology of 3D printed scaffolds. (E–H) Pore size distribution within each scaffold as determined by SEM and imageJ. There was no statistically significant difference between the average pore size in silk scaffolds, the cartilage phase of biphasic scaffolds and the bone phase of biphasic scaffolds (*p* > 0.05). However, 3D printed scaffolds did show significantly larger average pore size then all other scaffold types (*p* < 0.05). (J) Elemental distribution within each scaffold as determined by EDX. bone phase showed significantly reduced nitrogen content compared to all other groups and significantly increased carbon content compared to all other groups. (J) Scaffold porosity as determined by the Archimedes method. Silk scaffolds and the cartilage phase of the biphasic scaffolds showed the 2 highest porosities which were nonsignificantly different (*p* > 0.05); whereas 3D printed scaffolds and the bone phase of the biphasic scaffolds showed significantly lower (*p* < 0.01) porosity. There was no significant difference (*p* > 0.05) between the porosity of the 3D printed scaffolds and the bone phase of the biphasic scaffolds. Data represent mean ± SD.

Visual analysis of scaffolds demonstrated that biphasic scaffolds (Figure 2B) possessed a combined appearance of that of silk scaffolds (Figure 2A) and 3D printed scaffolds alone (Figure 2C)

SEM analysis showed that the biphasic scaffolds have two distinct regions. The cartilage phase (silk layer) showed a thin sheet-like network of lamellae with interconnected porosity (Figure 3A), which continued into the bone phase with no apparent change in silk morphology, with it successfully casting around the 3D printed lattice (Figure 3B). The silk component of the biphasic scaffold showed no noticeable morphological differences from the silk scaffolds alone (Figure 3C). Both biphasic and silk scaffolds showed a wide distribution of pore sizes ranging from 15 to 370 μm (Figure 3E–H). No significant difference (*p* > 0.05) was seen in the mean pore size of the cartilage phase (113 ± 52 μm), bone phase (124 ± 44 μm), and silk scaffolds alone (103 ± 51 μm). All scaffolds showed significantly (*p* < 0.05) smaller pore size than 3D printed scaffolds alone (768 ± 28 μm) (Figure 3H).

EDX analysis showed scaffolds’ elemental distribution as well as the presence or absence of any elemental contamination introduced during fabrication. This investigation indicated that carbon, oxygen, nitrogen, and gold were the only elements found within the 3D printed scaffolds, biphasic scaffolds, and silk control scaffolds. The presence of gold was associated with the sputter coating process required to visualize biological materials within the SEM due to its electrical conductivity. As no other elements were seen this indicated that no elemental contaminants were introduced into the scaffold during fabrication, such as lithium, bromide or sodium used during the silk purification process. Further investigation into the normalized weight distribution of carbon, nitrogen and oxygen (Figure 3I) between scaffolds showed that the cartilage phase of the biphasic scaffolds, which have the distribution of 45.15 ± 1.39% carbon, 20.35 ± 0.83% nitrogen and 34.50 ± 0.95% oxygen; this was not significantly different (*p* > 0.05) to the silk control scaffolds witch had a distribution of 45.67 ± 1.40% carbon, 21.67 ± 0.31% nitrogen and 32.65 ± 1.26% oxygen. These two scaffolds were compared to the bone phase of the biphasic scaffolds, which showed a significant increase (*p* < 0.05) in carbon 51.06 ± 1.89%, a significant decrease (*p* < 0.05) in nitrogen 15.24 ± 1.13%, and no significant change (*p* > 0.05) in oxygen content 33.69 ± 0.77% compared with the cartilage phase of the biphasic scaffolds and silk control scaffolds. On the other hand, the 3D printed scaffolds showed significantly higher (*p* < 0.001) carbon levels of 65.51 ± 0.31%, as well as significantly lower (*p* < 0.001) nitrogen 1.49 ± 0.10%, with no significantly different (*p* > 0.05) oxygen levels 33.00 ± 0.21% when compared with all other groups.

Scaffold porosity was evaluated using the Archimedes method (Figure 3J). Silk control scaffolds and the cartilage phase of the biphasic scaffolds showed the two highest porosities, with 90.03 ± 1.8% and 90.93 ± 2.7%, respectively, which were not significantly different (*p* > 0.05). In contrast, the 3D printed scaffolds and the bone phase of the biphasic scaffolds showed significantly lower (*p* < 0.01) porosities of 56.99 ± 0.4% and 61.01 ± 1.63%, respectively. There was no significant difference (*p* > 0.05) between the porosity of the 3D printed scaffolds and the bone phase of the biphasic scaffolds.

### 3.2 Mechanical Characteristics of the Biphasic Scaffolds

**Figure 4:**
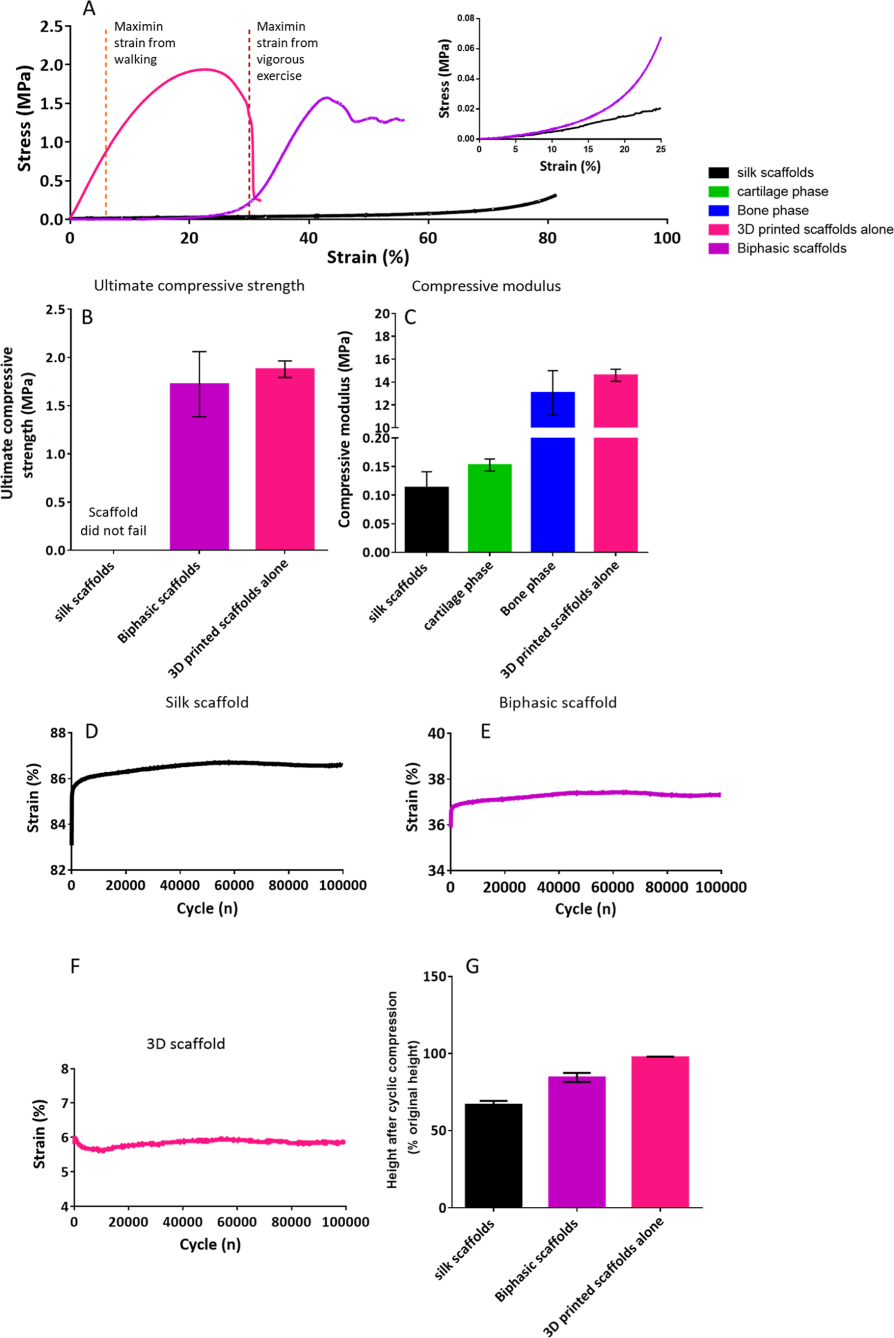
Scaffold mechanical properties (A) representative stress–strain curves for the scaffolds under uniaxial compression testing (B) ultimate compressive strength under uniaxial compression testing as determined via stress–strain curves. No statistically significant difference in ultimate compressive strength between biphasic scaffolds and 3D-printed control scaffolds (*p* > 0.05). (C) Scaffold compressive modulus under uniaxial compression testing as determined via stress–strain curves. Silk scaffolds and the cartilage phase of the biphasic scaffolds showed the 2 lowest compressive moduli, which were nonsignificantly different (*p* > 0.05), whereas 3D printed scaffolds and the bone phase of the biphasic scaffolds showed significantly higher (*p* < 0.001) compressive modulus. There was no significant difference (*p* > 0.05) between the compressive modulus of the 3D printed scaffolds and the bone phase of the biphasic scaffolds. (D–F) Fatigue testing shows the resultant strain every 100 cycles after applying a force of 8 N over 100,000 cycles. (D) Silk, (E) biphasic, (F) 3D printed. Silk control scaffolds significantly increased (*p* < 0.05) from the resultant strain at cycle one to cycle 100. Biphasic scaffolds also showed a significant increase (*p* < 0.05) between the resultant strain at cycle 1 to cycle 100; after cycle 100, no further significant (*p* > 0.05) changes were seen for both scaffolds compared to the nonsignificant change (*p* > 0.05) between the resultant strain at cycle 1 to cycle 100 seen in the 3D printed scaffolds. The decrease within the silk control scaffolds at cycle 100 was significantly greater (*p* < 0.05) than the decrease within the biphasic scaffolds. (G) Percentage remaining height of scaffolds after 100,000 cycles of fatigue testing at a load of 8 N. All scaffolds showed significantly different heights after fatigue testing (*p* < 0.001). Data represent mean ± SD.

A typical compressive stress–strain curve is shown in Figure 4A. The 3D-printed lattice provides rigidity and a greater load resistance than the silk scaffold control. The compressive modulus was significantly greater (*p* < 0.001) in the bone phase of the biphasic scaffold group (12.56 ± 1.94 MPa) compared to that of the silk scaffolds (0.113 ± 0.028 MPa) (Figure 4C). However, there was no significant difference (*P* > 0.05) between the silk scaffold and the cartilage phase of the biphasic scaffold (0.12 ± 0.01 MPa). There was also no significant difference (*P* > 0.05) between the bone phase of the biphasic scaffold and the 3D printed scaffold (14.60 ± 0.53 MPa). The incorporation of the silk layer appears to have no detrimental effects on the biphasic scaffold’s ultimate compressive strength, demonstrated by there being no significant difference (*P* > 0.05) on the ultimate compressive strength between the 3D printed scaffold (1.88 ± 0.087 MPa) and the biphasic scaffold (1.56 ± 0.34 MPa) (Figure 4B). The silk top layer on the biphasic scaffold also significantly increased the strain at failure from the 3D printed scaffold (25.7 ± 4.5%) to the biphasic scaffold (42.1 ± 7.3%) (*p* < 0.001). The biphasic scaffold retrieved at the end of compression testing consisted of a flattened scaffold. There was no sign of delamination between phases. The presence of silk appeared to increase the resilience of the scaffold dramatically, extending the toe region, as seen in Figure 4A.

### 3.3 Fatigue Behavior of the Biphasic Scaffolds

During fatigue testing, the biphasic scaffolds and silk control saw an increase in resultant strain after the first 100 cycles, followed by a plateau with very little further change (Figure 4D,E) in contrast with the 3D-printed scaffold alone, which saw a slight decrease in resultant strain.

Biphasic scaffolds showed a significant increase (*p* < 0.05) between the resultant strain at cycle 1 (35.88 ± 0.69%) to cycle 100 (36.55 ± 0.64%) followed by no further significant changes. Silk control scaffolds also presented a significant increase (*p* < 0.05) in the resultant strain at cycle one (83.08 ± 1.92%) to cycle 100 (85.13 ± 1.60%) followed by no further significant changes. This is in comparison to the nonsignificant change (*p* > 0.05) between the resultant strain at cycle 1 (5.81 ± 0.58%) to cycle 100 (5.99 ± 0.60%) seen in the 3D printed scaffolds (Figure 4F). However, the decrease seen over the first 100 cycles within the silk control scaffolds (2.05 ± 0.34%) was significantly greater (*p* < 0.05) than the decrease seen within the biphasic scaffolds (0.67 ± 0.11%).

Following 100,000 cycles, samples were evaluated for change in overall height as a measure of permanent damage to the scaffolds (Figure 4G). All scaffolds showed a significantly (*p* < 0.05) decreased height after 100,000 cycles of fatigue testing compared to their starting height with all decreased heights being significantly different (*p* < 0.001) between groups. Biphasic scaffolds saw a reduction of 18.5 ± 2.5%, silk scaffolds showed a reduction of 33.9 ± 1.5% and the 3D printed scaffold alone saw a reduction of 2.4 ± 0.5%.

### 3.4 Swelling and Degradation of the Scaffolds

**Figure 5:**
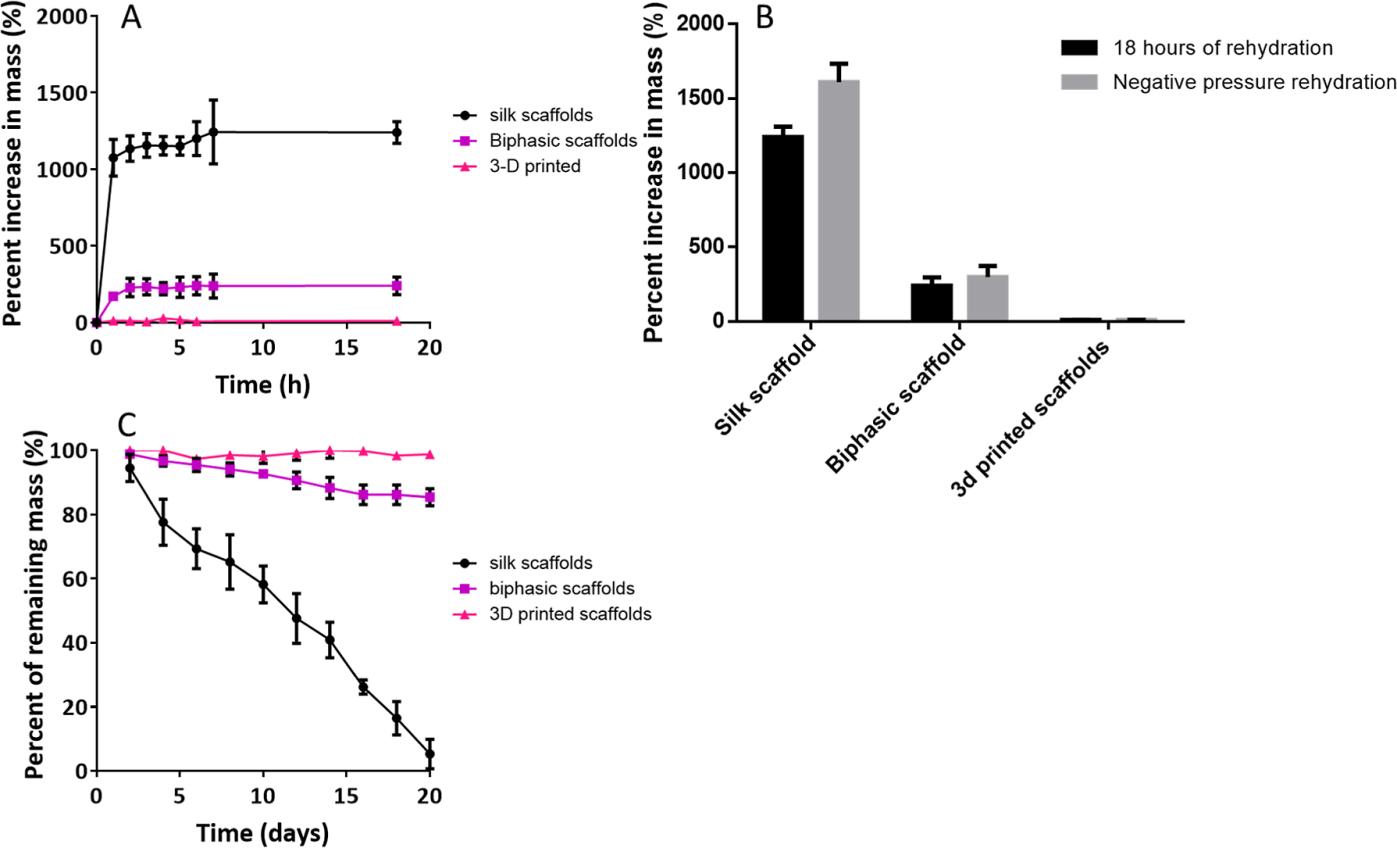
Scaffold swelling and degradation behavior. (A) Swelling potential of scaffolds over 18 h. All percentages of mass changes after the first hour were significantly different (*p* < 0.0001). Following the initial increase within the first hour, all three scaffolds saw no significantly different further changes in mass in the subsequent 18 h (*p* > 0.05). All changes in mass were significantly different between groups at all time points (*p* < 0.001). (B) Comparison of scaffold rehydration after 18 h of rehydration in 1× PBS with rocking or 18 h of rehydration plus negative pressure rehydration. Silk scaffolds and biphasic scaffolds showed a significant increase in percentage mass increase (*p* < 0.01), whereas the 3D-printed scaffolds saw no significant change (*p* > 0.05). (C) In vitro degradation of scaffolds submerged in protease solution over 20 days. Initially, silk scaffolds, biphasic scaffolds and 3D printed scaffolds showed similar degradation rates, with after 2 days, there being no significant difference (*p* > 0.05) in mass decrease. However, in all the following days, the silk scaffolds had a more significant decrease in mass than the biphasic scaffolds and 3D-printed scaffolds. After 20 days, all scaffolds showed a significant difference in mass. Data represent mean ± SD.

Both biphasic scaffolds and silk scaffolds showed an initial rapid increase in mass after 1 h, with biphasic scaffolds undergoing an increase of 172 ± 19% and silk scaffolds seeing an increase of 1075 ± 103% and the 3D printed scaffolds seeing a small increase of 12 ± 4.7% (Figure 5A). Following the initial increase within the first hour, all three scaffolds saw no significantly different further changes in mass in the subsequent 18 h (*p* > 0.05). All changes in mass were significantly different between groups at all time points (*p* < 0.001). Following the initial increase within the first hour, all scaffolds demonstrated minimal further changes in mass and the subsequent 18 h.

To confirm whether the scaffolds were fully rehydrated after 18 h of incubation in 1× PBS, a negative pressure rehydration step was conducted. This consisted of placing the scaffolds within a low-pressure environment while they were submerged in PBS. Both the silk control scaffolds and the biphasic scaffolds saw significant increases in mass compared to the 18 h level after negative pressure rehydration (*p* < 0.01). Whereas the 3D printed scaffolds saw no increase in mass (Figure 5B). Biphasic scaffolds increased from an 18 h level of 240 ± 47% to a post negative pressure rehydration level of 299 ± 62% (*p* < 0.01). Silk control scaffolds increased from the 18 h level of 1241 ± 61% to the postnegative pressure rehydration level of 1611 ± 106% (*p* < 0.01). However, the 3D-printed scaffolds saw no change between the 18 h level of 11 ± 1.4 and the postnegative pressure rehydration level of 11 ± 2.6 (*p* > 0.05). This indicates that passive diffusion of fluid into the biphasic and silk scaffolds was not satisfactory to fully rehydrate the scaffolds, and negative pressure rehydration is required for complete scaffold rehydration. This was further visually confirmed by the scaffolds transitioning from floating when placed within liquid to sinking after negative pressure rehydration.

Initially, silk scaffolds, biphasic scaffolds and 3D printed scaffolds showed similar degradation rates; after 2 days, there was no significant difference (*p* > 0.05) in mass decrease, with silk scaffolds having 94.5 ± 4.2%, biphasic scaffolds having 98.8 ± 1.1% and 3D printed scaffolds having 99.3 ± 1.1% of the original mass (Figure 5C). After 20 days, all scaffolds showed a significant difference in mass to each other whereby the silk scaffolds had 5.3 ± 4.6% of the scaffolds’ starting mass, the biphasic scaffolds had 85.4 ± 2.6% and the 3D printed scaffolds had 98.8 ± 0.6%.

### 3.5 Cells Viability

**Figure 6:**
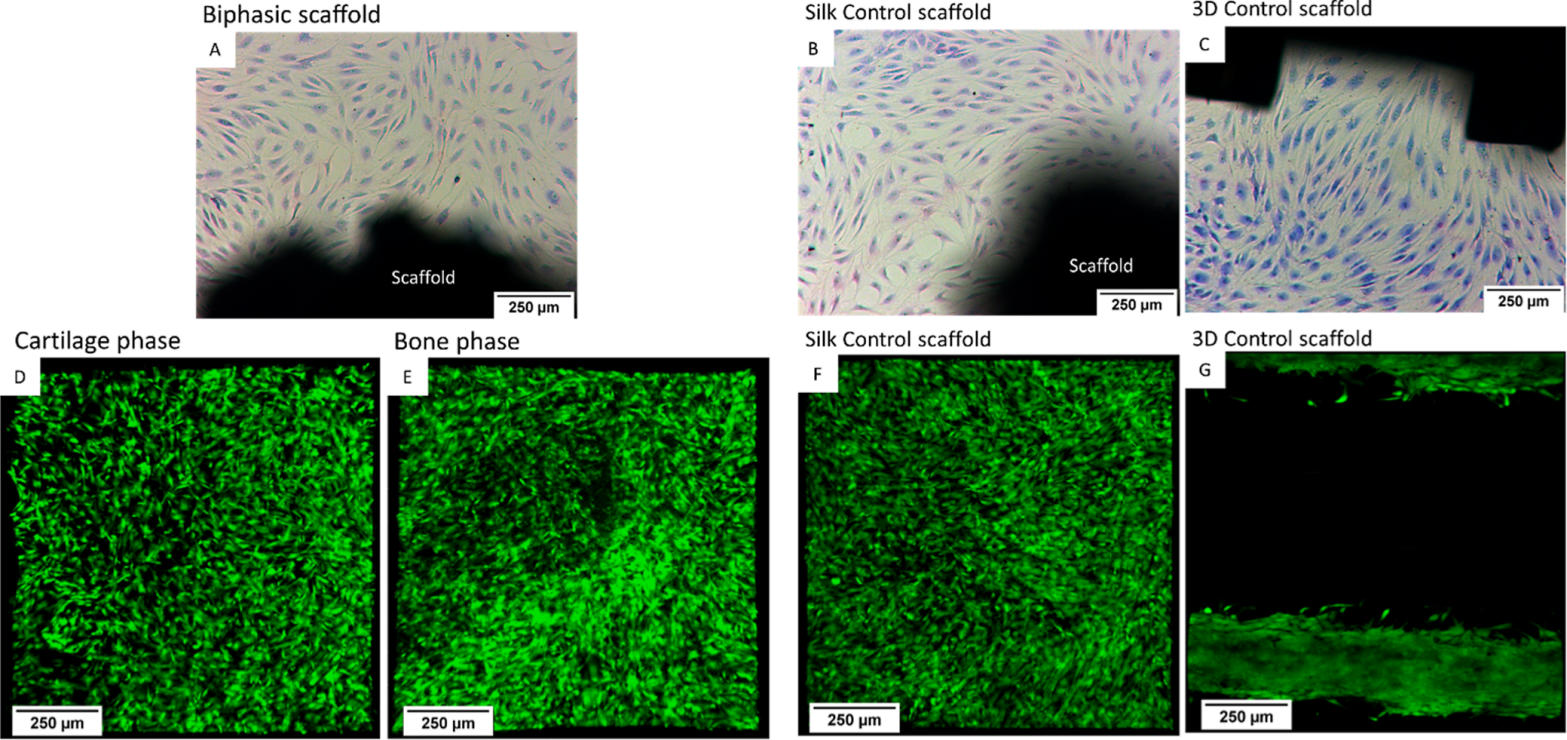
Cell scaffold interactions (A–C) Investigation of contact cytotoxicity via Giemsa staining, as per ISO10993-5:2009 (E) of hMSC-BM cultured in the presence of biphasic scaffolds (A), silk control (B) and 3D printed control (C). (D–G) Confocal images of hMSC-BM labeled with CMFDA labeled seeded on silk scaffolds, 3D printed and biphasic after 24 h of culture. Cartilage phase of biphasic scaffold (D), bone phase of biphasic scaffold (E), silk scaffolds (F) and 3D printed (G).

Contact cytotoxicity was undertaken as per ISO 10993-5:2009; the results indicate all three scaffold types showed no signs of contact cytotoxicity as evidenced by no cytotoxic zone being seen around any scaffold type (Figure 6A–C). The hMSC-BM in all the groups showed normal cell morphology, with good membrane integrity and no cell detachment or lysis. Cell viability and morphology after short-term seeding were evaluated by seeding of hMSC-BM onto all three scaffold types and visualized with CMFDA labeling. The confocal laser scanning micrographs revealed a high proportion of viable green cells in all scaffold types. There were no observable differences in viability across all groups at 24 h (Figure 6D–G). However, silk scaffolds, the cartilage phase of biphasic scaffolds and the bone phase of biphasic scaffolds showed a much more even distribution of cells across the surface of scaffolds compared to 3-D printed scaffolds, where cells were limited and restricted the filaments of the 3D printed lattice leading to large regions with no cells.

## 4 Discussion

Due to osteochondral tissue’s hierarchal and complex architecture, scaffolds representing cartilage and bone may be required for proper and satisfactory osteochondral regeneration.^25^ However, osteochondral tissue comprises two unique tissue types (bone and cartilage), so regeneration of this tissue is particularly challenging. This study designed a scaffold to tackle this challenge by combining two well established biomaterials and deploying them into a novel biphasic scaffold in a unique way. This unique biphasic scaffold consisted of a bone phase made up of a 3D printed poly(ethylene glycol)-terephthalate-poly(butylene terephthalate) lattice in which silk was utilized as an infill material which was continuously blended to a porous silk only cartilage phase. This scaffold was then characterized for its physical and mechanical properties, allowing for conclusions to be drawn about its potential future use and deployment in osteochondral tissue regeneration.

The flexibility of 3D printing technology is a significant advantage in the field of tissue engineering, as it allows for the fine-tuning of many fundamental parameters for creating scaffolds, such as scaffold pore size and mechanical behavior, due to the ability to adjust printing parameters like nozzle diameter, print speeds, temperatures, and feed rates. Additionally, it enables the creation of geometries that would otherwise be impossible to fabricate. Furthermore, 3D printing fits well within the ever-growing field of personalized medicine, offering the potential for scaffolds to be tailored to an individual’s specific tissue engineering requirements. For instance, in the field of osteochondral tissue engineering, varying the shape and size of scaffolds can be crucial for addressing individual osteochondral defects. However, this technology still presents notable drawbacks. For example, the large pores in some 3D-printed scaffolds can cause difficulties in cell bridging.^14^ Therefore, there is potential for using secondary infilling materials to enhance the porosity of 3D-printed scaffolds while still maintaining their versatility.

Pore size and porosity are integral to the scaffold’s function, as these two factors dramatically affect cell adhesion, proliferation, differentiation, ingrowth, and the transportation of nutrients and waste products. Porosity and pore size also play a fundamental role in angiogenesis and revascularisation in vivo.^15,26^ When designing a scaffold with an optimal pore size for osteochondral tissue regeneration, the difficulty is often seen in that both subchondral bone and cartilage scaffolds have differing optimal pore sizes.^27^ Studies have previously demonstrated that for optimal cartilage regeneration a pore size of approximately 90 to 120 μm is desirable as this helps to direct and encourage chondrogenesis^28,29^ It has been previously shown that optimal scaffold pore size for subchondral bone regeneration is a larger size than that seen for chondrogenesis (approximately 300 μm), as this pore size seems to favor direct osteogenesis while also allowing for vascularisation, providing a relatively high oxygenation within the scaffold. However, smaller pores do still allow for osteogenic differentiation and bone formation, but this is only following cartilage formation.^15,28,30,31^ Previous attempts to utilize a synthetic 3D printed lattice for tissue regeneration have demonstrated an issue with the lattice structure; due to the large pore size seen in the lattice structure, cells often struggle to migrate between the filament’s large gaps, retarding the migration of cells.^14^ The larger pore size also demonstrates the problem of reduced cell adhesion due to the lower availability of specific areas for ligand binding.^32^ The cartilage phase of biphasic scaffolds demonstrated a pore size of 117 ± 15 μm that falls within the optimal range, as previously demonstrated in the literature, of approximately 90 to 120 μm for chondrogenesis.^28,33^ This is compared to the bone phase where by utilizing a silk-infilling material within the bone phase of biphasic scaffolds, the pore size could be functionally reduced to 124 ± 24 μm compared to that seen within 3D printed lattices alone of 768 ± 28 μm. The reduction of pore size increases the ligand binding surface area for cells and the surface area for extracellular matrix deposition. The ability of a silk infill material to improve cell binding was reinforced by hMSC-BM showing equivalent adherence on silk scaffolds and biphasic scaffolds as compared to 3D printed scaffolds, where cells were limited to the filaments of the 3D printed lattice. The silk infilling was demonstrated to be an effective technique to improve cell adherence and migratory potential for scaffolds incorporating 3D printed components. However, the reduced pore size presents a disadvantage as it is lower than the recommended pore size for osteogenesis and vascularisation of 300 μm. However, due to the natural nature of the silk component, it is theorized degradation occurs more rapidly in vivo while the synthetic 3D component remains; this provides the notable advantage of degrading to allow for vascularisation but this still maintaining the structural protection the 3D printed component provides to the newly formed tissue within the bone phase. The in vitro data supports this theory as showed that the proteolytic solution preferentially degraded the silk component of biphasic scaffolds, leaving the 3D printed component relatively unchanged, indicating its greater resistance to degradation, which was assigned to its synthetic nature being dramatically less susceptible to proteolytic degradation.

Within this study, the biphasic scaffold showed dramatically less degradation compared to the silk control scaffolds. The silk control scaffolds exhibited similar degradation profiles to other studies that investigated silk-only scaffolds.^34^ The 3D-printed component of the biphasic scaffolds showed greater resistance to degradation than the silk infill material, as confirmed by visual inspection, showing almost complete degradation of the infilling silk material within the biphasic scaffold after 20 days. The 3D-printed component appeared to provide a small amount of protection against the degradation of the infilling silk material, as the remaining mass seen within the biphasic scaffold of 85.4 ± 2.6% is approximately 5% higher than what would be expected. Bearing in mind, that there was 5.3 ± 4.6% of the silk-only scaffold’s mass remaining after 20 days, and the weight ratio of silk infilling material to the 3D-printed component is 1:4. This protective ability was attributed to the 3D-printed component diminishing the surface area available for degradation of the infilling silk material. Silk biodegradation in vivo is primarily mediated by proteolytic enzymes such as matrix metalloproteinases (MMPs), and lysosomal proteases (cathepsins). As these enzymes can cleave the peptide bonds in silk proteins.^35−38^ Previous studies that investigated silk fibroin degradation in vitro showed similar in vitro degradation rates as the scaffolds seen within this study.^34^ The same study also showed in vivo that silk fibroin scaffolds lose about 50% of their original size within 12 weeks. Further studies have also shown that scaffolds implanted subcutaneously have a tendency to be completely degraded within six months.^39^ The degradation rate during subcutaneous implantation appears to be similar to that seen when silk scaffolds are implanted within articular cartilage. With scaffolds seeing a large amount of degradation and replacement with natural tissue within 12 weeks and complete replacement with native tissue after six months.^40,41^ Heavily inferring that the scaffolds created within this study are most likely to have an adequate degradation rate that matches osteochondral tissue regeneration. Although further in vivo studies will need to be undertaken to confirm this.

Within this study, the cartilage phase of the biphasic scaffold showed a comparable porosity to the silk control scaffolds, with a porosity of 90.93 ± 2.7% compared to that of silk scaffolds alone (90.03 ± 1.8%). It has been indicated that porosity of greater than 70% is suitable for tissue regeneration,^15,16^ as this amount theoretically allows for cell infiltration into the scaffold surface and adequate permeability for oxygen, nutrients and waste exchange. However, the bone phase of the biphasic scaffolds showed a less than 70% porosity of 61.01 ± 1.63%; this is comparable to the 3D printed scaffolds (56.99 ± 0.4%). The reason for the reduced porosity was assigned to the nonporous nature of the 3D-printed filaments found within the bone phase taking up a large proportion of this phase. Although the porosity of the bone phase is less than the indicated 70%, this fails to consider the nuances of the scaffold design, as the silk component of the bone phase is most likely to have a high comparable porosity to a silk control scaffold. Thus, although the bone phase of the biphasic scaffold as a whole has less than 70% porosity, the regional variability of the bone phase should mean that there should still be adequate potential for cell infiltration as well as nutrient, oxygen, and waste exchange within the bone phase of the biphasic scaffolds via the silk component.

The ability of sponge-like scaffolds to swell and retain water/liquid within their structure is essential for their regenerative capacity and potential cellular interactions.^42,43^ A scaffold’s failure to rehydrate can fundamentally reduce its effectiveness regarding tissue engineering and its ability to regenerate natural tissue. Failure of rehydration can lead to the collapse of scaffold pores, reducing porosity,^44^ affecting cell penetration and reducing nutrient and waste exchange through the scaffold’s interconnected pore network. Furthermore, lacking an aqueous environment can reduce cell adhesion to the scaffold surface. The swelling capacity of the scaffolds investigated in this study differed based on the material composition, with the biphasic scaffolds showing a lower capacity than the silk control scaffolds. This difference in swelling capacity was assigned to the 3D printed components of the biphasic scaffold’s limited swelling capacity and its considerable contribution to the scaffolds’ initial mass. It was also found that passive liquid diffusion into the scaffold could not fully rehydrate scaffolds from the dried state. Hence, a negative pressure rehydration step is required to rehydrate the scaffolds completely. The reason for the lack of complete rehydration under passive diffusion conditions was assigned to the surface tension of the liquid, limiting the depth at which liquid could penetrate through pores to the center of scaffolds. However, this study determined that the simple method of negative pressure rehydration could be used to entirely rehydrate silk and biphasic scaffolds. Negative pressure rehydration was conducted by first placing scaffolds into liquid before lowering the relative atmospheric pressure surrounding the scaffold. This caused the air bubbles enclosed within the scaffold’s pores to be removed, and the pores were then filled with the liquid in which the scaffold was submerged. The negative pressure rehydration step utilized within this study demonstrates an easy and effective way to induce scaffold rehydration within a relatively short time frame. It should enable an increased potential cell infiltration and an increased regenerative capacity.

The mechanical properties of the scaffold are fundamental to its regenerative capacity; its mechanical properties need to be great enough to resist articulation and manipulation during implantation but not so great that they stress shield mechanosensitive cells. The selection to blend two material types for the bone phase enables the scaffold to possess improved characteristics for osteochondral tissue regeneration. Silk scaffolds have been shown to have excellent biocompatibility.^34,45,46^ However, they have also been shown to have weak mechanical properties making them difficult and undesirable in load-bearing applications. Therefore, as this study has demonstrated, using a synthetic 3D-printed lattice infilled with silk can improve the scaffold’s mechanical properties. The addition of the 3D-printed lattice increases the scaffold’s long-term survival and regenerative capacity. The ultimate compressive strength of the bone phase within the biphasic scaffold (1.465 MPa) appears to be high enough to allow the scaffold to survive implantation and joint loading.^47^ The seamlessly integrated silk layer further enables the scaffold to represent native tissue, as the cartilage phase could undergo a large amount of deformation while still maintaining shape recovery. It has previously been shown that under normal walking, cartilage undergoes a maximum strain of no greater than 6%, and a maximum strain of no greater than 30% during vigorous exercise.^48−51^ The biphasic scaffolds used in this study demonstrate a strain at failure of 42.1 ± 7.3%, which is well above the 30% seen during vigorous exercise and well before the yield point of the scaffold. This indicates that the synthesized biphasic scaffold should have adequate properties to survive physiologically relevant strains post implantation.

Rather than implanted scaffolds experiencing overloading forces, it is much more likely that they will experience low-intensity repeated fatigue loading during normal articulation.^50^ It has previously been shown that cartilage is loaded at approximately 1 Hz during normal walking, with a strain-no greater than 6%.^48−50^ As demonstrated, all three scaffolds can survive repeated loading far over the strain seen within normal articular cartilage of 6% with no major failure on any scaffolds. 3D printed control scaffolds showed the greatest propensity to resist fatigue loading showing very little change in the scaffold’s height (2.4 ± 0.5%). This is in contrast to silk control scaffolds showing the least propensity to resist fatigue loading with the greatest reduction (33.9 ± 1.5%). As expected, the biphasic scaffolds showed a blend of fatigue behavior of the two scaffolds (18.5 ± 2.5%). This data demonstrates that, theoretically, the biphasic scaffolds within this study can survive low-intensity repeated loading postimplantation.

Although the biphasic scaffolds fabricated in this study can theoretically withstand the physiological forces experienced in a joint, their mechanical properties still fall below those of native osteochondral tissue. Previous studies have shown that native articular cartilage has a compressive modulus in unconfined uniaxial compression testing between 0.34 and 1.202 MPa, compared to 0.12 ± 0.01 MPa for the cartilage phase of the biphasic scaffold fabricated in this study.^52,53^ Additionally, native subchondral bone has a compressive modulus between 297 and 475 MPa, as reported by previous studies under unconfined uniaxial compression testing.^54^ This contrasts with the bone phase of biphasic scaffolds in this study, which exhibited a compressive modulus of 14.60 ± 0.53 MPa. However, scaffolds’ bulk mechanical properties and survivability postimplantation are not the only factors to consider for a scaffold’s mechanical properties. It has been extensively shown that scaffold stiffness can dramatically impact the differentiation or capacity of hMSC-BM.^55−61^ Scaffold mechanical properties are crucial in influencing cell behavior and tissue regeneration. The stiffness of scaffolds significantly affects the differentiation of hMSC-BM. A number of studies showed that stiffer substrates direct hMSC-BM toward osteogenic lineage, while softer substrates promote adipogenic differentiation, with an intermediate stiffness favoring chondrogenic differentiation. Optimal stiffness for osteogenic differentiation ranges from 40-100 kPa, while chondrogenic differentiation may require a substrate stiffness of 10–50 kPa.^61−76^ The biphasic scaffolds in this study consist of a 3D-printed lattice infused with silk. Although the bulk compressive modulus of the bone phase is much higher (12,560 kPa) then the optimal range for osteogenic differentiation, cells are more likely to experience a stiffness closer to that of the silk control scaffolds (113 kPa) due to the prominence of the silk infill material. This still falls slightly above the optimal range for osteogenic differentiation but remains effective for bone regeneration. As for chondrogenic differentiation, the cartilage phase of the biphasic scaffolds has a stiffness of 152 kPa, witch is higher than the optimal range for chondrogenic differentiation. However, the impact of growth factors and signaling molecules seems to influence hMSC-BM differentiation toward chondrogenic lineage more than substrate stiffness.^75^

Some previous studies have also investigated the utilization of an infilling material in 3D-printed scaffolds to create a composite design for tissue engineering. However, the majority of these studies focus on the utilization of hydrogels rather than porous scaffolds, and mostly on the scaffold’s cellular interactions rather than its mechanical or physical properties.^77−80^ Li et al. demonstrated that a hydrogel consisting of a self-assembled peptide infused within a PCL 3D-printed scaffold was able to induce an improved healing response within an osteochondral defect in a rabbit model. Furthermore, the potential of this technology was highlighted by Wang et al., who combined freeze-dried porous scaffolds with hydrogels and 3D-printed scaffolds also showing success in improving osteochondral tissue regeneration within an animal model. The current study provides valuable mechanical and physical context to the existing literature supporting the potential of infilling materials to enhance the regenerative capacity of 3D-printed scaffolds. It highlights and provides mechanical context to the protective characteristics of the 3D-printed component in relation to the biologically active infilling material, offering insights into how combining these elements can create a versatile scaffold for potential application across various tissues. In particular, the freeze-dried porous scaffold used in this study, designed for osteochondral tissue regeneration, demonstrates promising mechanical and physical properties for a regeneration of this tissue type. By utilizing materials beyond hydrogels, such as porous scaffolds, this approach could help mitigate some limitations associated with hydrogels, like poor cellular mobility. Although the physical and mechanical properties of the scaffold suggest its suitability for osteochondral regeneration, further in vivo studies and in vitro animal models are needed to confirm its osteogenic and chondrogenic potential.

## 5 Conclusions

In this study, a novel biphasic scaffold was created by combining a of 3D printed PEGT/PBT lattice with *B. mori* silk fibroin. The 3D printed component within the scaffold provided a solid framework that increased the versatility and provided a mechanically robust structure that can theoretically survive the forces seen during joint articulation while improving the degradation profile. The silk infilling provided the secondary porous structure to the 3D-printed scaffold for the bone phase and a superficial layer for the cartilage phase. Silk within both phases improved the scaffold’s biocompatibility and cell adhesion characteristics, increasing the scaffold’s surface area. This unique biphasic 3D printed silk-infilled scaffold has the potential to fill a niche within osteochondral tissue regeneration, especially with the possibility for its use within personalized medicine, with the 3D printing structure easily being adapted to different individuals. Although these results are highly promising for the scaffold's future use in osteochondral tissue regeneration, further *in vivo* and *in vitro*characterization needs to be undertaken to confirm its capacity to direct and support osteochondral differentiation.
